# *Salmonella* in pigs slaughtered in Ecuador: prevalence, serotypes, genotypes and antibiotic resistance

**DOI:** 10.1016/j.vas.2025.100548

**Published:** 2025-11-22

**Authors:** M. Cevallos-Almeida, C. Gómez, B. Cajas, A. Almachi, V. Rose, M. Denis, A. Kerouanton

**Affiliations:** a Laboratory of Bacteriology and Mycology. Faculty of Veterinary Medicine and Zootechnics. Central University of Ecuador 170521 Quito, Ecuador; b French Agency for Food, Environmental and Occupational Health and Safety, ANSES, Ploufragan-Plouzané-Niort Laboratory, Hygiene and Quality of Poultry and Pig Products Unit 22440 Ploufragan, France

**Keywords:** *Salmonella*, Pig, Slaughterhouse, PFGE, Antimicrobial resistance, Serotype

## Abstract

*Salmonella enterica* subsp. *enterica* (*S. enterica*) is a leading foodborne pathogen worldwide, and pork products are major sources of infection. Given the lack of data on *S. enterica* infection in pigs slaughtered in Ecuador, this study aimed to determine its prevalence, as well as the serotypes, genotypes and antimicrobial susceptibility profiles of isolates from cecum contents (CCs) and mesenteric lymph nodes (MLNs) sampled from 365 pigs at the evisceration step at a slaughterhouse in Quito city. *Salmonella* spp. in the samples was detected by culture (ISO 6570–1 method). Strains were serotyped (ISO 6579–3 method), tested for antimicrobial susceptibility using the disk diffusion method, and genotyped by macrorestriction of chromosomal DNA. *Salmonella enterica* was detected in 56 of the 365 pigs sampled (15.3 %), in CCs (11.2 %) or MLNs (11.2 %) but never in both. The 56 isolates (one per positive sample) belonged to 12 serotypes, mainly the monophasic Salmonella Typhimurium (mST) (28.5 %) and *S*. Uganda (26.7 %). Twenty-two *Xba*I PFGE profiles were identified, with higher genetic diversity for *S*. Infantis and *S*. Uganda. A high percentage of isolates showed antimicrobial resistance to tetracycline (71.4 %) and streptomycin (62.5 %). Twelve antimicrobial multi-resistant patterns were observed, particularly associated with S. Typhimurium isolates, *S*. Infantis and mST. The present study constitutes the first investigation of *S. enterica* prevalence in a large-scale slaughterhouse in Ecuador. The findings will contribute to a better assessment of the epidemiology of *S. enterica* in the pork industry and of the associated public health risk.

## Highlights

• First prevalence study of *salmonella* at pigs slaughterhouse level in Ecuador.
• Higher *Salmonella* recovery in cecal contents compared to mesenteric lymph nodes.
• No simultaneous recovery from both samples in the same pig.
• Majority of serotypes commonly associated worldwide with pig production and implicated in human salmonellosis.
• Multidrug resistance detected in 18 isolates, including 4 ESBL-producers.

## 1 Introduction

*Salmonella enterica* subsp. *enterica* (*S. enterica*) is one of the four leading causes of diarrheal diseases worldwide, with >2600 serotypes (Balasubramanian et al., 2019; Lamichhane et al., 2024). Most *Salmonella* outbreaks in humans are associated with the consumption of eggs, poultry and beef, but, over the last two decades, a significant proportion has been traced to the consumption of pork and its products (Ehuwa et al., 2021). In Latin America, the swine industry is an important industry that contributes significantly to the economies of these regions (FAO, 2019; USDA, 2023; Roppa et al., 2024). As an example, in 2020, Ecuador had around 40,000 sows and a production of 170,000 tons of pork (Ionita, 2022).

Pigs host *Salmonella* in the intestinal tract and are mainly asymptomatic carriers and intermittent shedders. *Salmonella* can also colonize the pig lymph nodes or tonsils, making them a potential source of carcass contamination during slaughter (Soliani et al., 2023). Several studies have demonstrated that pigs arriving at slaughterhouses have a higher prevalence of *Salmonella* than on the farm of origin, and that the prevalence of *Salmonella*-infected pigs entering the slaughtering process has an impact on the levels of contamination in the slaughterhouse environment (Bernad-Roche et al., 2022; Berriman et al., 2013; Martelli et al., 2021). In southern Brazil, the overall prevalence of *Salmonella* in pig slaughterhouses has been found to be 14.8 % (40/270), with *Salmonella* detected mainly in feces (Bersot et al., 2019). Studies in Colombia and Peru have reported *Salmonella* prevalence in pig carcasses of 16.7 % and 6.3 %, respectively (Carrascal-Camacho et al., 2023; Salvatierra et al., 2015).

The pig slaughtering process is a critical stage in the spread of *S. enterica*, due to the cross-contamination that can occur during the different steps, from before slaughter to the final processing of the meat. Slaughtering practices and hygienic conditions in slaughterhouses play a major role in the prevalence of *S. enterica* in meat products intended for human consumption (Soliani et al., 2023). During the pre-slaughter phase, stress in pigs can increase *Salmonella* excretion, and pigs can become infected through cross-contamination during transport and lairage (Arguello, Álvarez-Ordoñez et al., 2013). In addition, in lairage pens, exposure of pigs to contaminated environments, even for short periods, can lead to infection of healthy pigs, with *Salmonella* present in the oral cavity, the tonsils and even the intestinal contents (Van Damme et al., 2018). Slaughter, evisceration, and the handling of internal organs are also critical stages in the spread of *S. enterica* because accidental rupture of the intestines of infected pigs is likely to cause massive contamination of the work area, the carcass and, consequently, the processed products (Viltrop et al., 2023).

The knives used for pig evisceration can also serve as vectors for *Salmonella* transmission, especially if they are not properly disinfected (Van Damme et al., 2018). The post-slaughter phase includes the cooling and storage of carcasses, during which contamination may persist if strict hygiene protocols are not followed. Inadequate handling of pig carcasses and the use of non-disinfected equipment have been identified as key factors contributing to the persistence of *Salmonella* in processing plants (Arguello, Sørensen et al., 2013; Viltrop et al., 2023).

Two important sites of *Salmonella* colonization in pigs are the mesenteric lymph nodes (MLNs) and the cecum (Cevallos-Almeida et al., 2018; Österberg et al., 2010). At slaughter, cuts in lymphatic tissues can lead to the contamination of pig carcasses by *S. enterica*. The presence of *Salmonella* in the MLNs of pigs from different origins and slaughterhouses, as well as the circulation of different serotypes and genotypes of *Salmonella* in different geographical regions, are of great concern and indicate that the pig production sector could be contributing to the spread of this pathogen (Possebon et al., 2020). Prior to this study, there were no data on *Salmonella* in pig slaughterhouses in Ecuador, and only a few data were available on the presence of *S. enterica* in pork products sold in retail outlets and public markets in the city of Quito (Mejia et al., 2021; Vinueza-Burgos et al., 2023).

Antimicrobial resistance in *Salmonella* is a widespread international concern and, since 1990, the occurrence of multidrug-resistant isolates of *S. enterica* has increased dramatically in many developed countries (Threlfall, 2002). In Latin America, the levels of antimicrobial resistance observed are low, which could reflect either reduced use of antimicrobials in farms or lack of surveys (Van Boeckel et al., 2019). However, in these countries, including Ecuador, antimicrobial resistance and multiresistance (up to 10) has been reported for *Salmonella* strains isolated from pigs and pork (Ibar, 2017; Mejía et al., 2020; Mejia et al., 2021; Vico et al., 2020; Vinueza-Burgos et al., 2019, 2023).

The aim of this work was to assess the prevalence of *S. enterica* in MLNs and cecal contents of pigs slaughtered in a major slaughterhouse in the city of Quito, Ecuador, and, to determine the serotype, the antimicrobial susceptibility, and the genotypic relatedness of the isolated strains.

## 2 Material and methods

### 2.1 Study design and sampling

This study was conducted in a major slaughterhouse in the city of Quito from November 2018 to June 2019. In the absence of any existing data on the occurrence of *Salmonella* contamination at slaughterhouses in Ecuador, we assumed a prevalence of 50 % with 5 % of sampling error, as recommended in veterinary epidemiological research (Noordhuizen et al., 2001; Stevenson, 2021). From this estimation and the average number of pigs slaughtered in this slaughterhouse every month (7000), we calculated a minimum total sample size of 365 animals with a 95 % confidence interval (CI) (Noordhuizen et al., 2001; Stevenson, 2021).

**Fig. 1:**
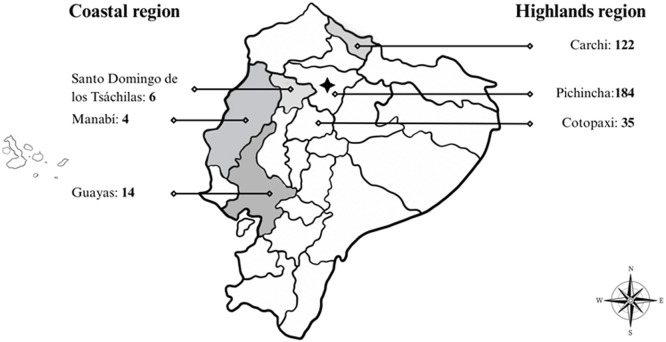
Geographical location of slauhterhouse (*) and state where the pigs originated (•) in Ecuador.The number of pigs sampled is indicated for each state.

Pigs slaughtered in this slaughterhouse came from two of the four regions of Ecuador: the coastal region (*n* = 24 pigs sampled), and the highlands region (*n* = 341 pigs sampled). In each region, pigs sampled had been raised in three different states (Guayas, Santo Domingo de los Tsachilas and Manabi in the coastal region and Carchi, Pichincha and Cotopaxi in the highlands region) (Fig. 1).

Animals were selected at random during the evisceration process and two types of samples were taken from each pig, *i.e.* caecal content (CC) and MLNs. A total of 10–20 pigs were sampled weekly for eight months. The viscera corresponding to the carcasses sampled were placed in conveyor trays. The surface of the cecum was disinfected using a cotton swab with ethanol 70 % and, after performing a small incision, 80 g of CC was collected in a sterile container. The mesentery from the same carcass was disinfected as described for the cecum and the MLNs from the small intestine (mainly at the jejunum or ileum) were collected in a sterile container. Samples were transported in cold conditions for further analysis at the Bacteriology and Mycology Laboratory of the Faculty of Veterinary Medicine and Zootechnics of the Central University of Ecuador.

### 2.2 *Salmonella* isolation

*Salmonella* spp. was isolated according to the ISO 6579–1 standard. Briefly, 25 g of sample was diluted 1:10 in buffered peptone water (BD Difco, USA) and then homogenized before being incubated at 37ºC for 18 h. Three drops of the initial suspension were then placed on Modified Semi-solid Rappaport Vassiliadis agar (BD Difco, USA) and incubated at 42 °C for 24–48 h. According to their growth, plates were classified into: growth and migration (GM), growth with no migration (GNM) and no growth (NG).

From the migration halo, all GM plates were streaked onto Xylose, Lysine, Deoxycholate medium (Difco, USA) and incubated at 37 °C for 24 h. For biochemical confirmation, two presumptive *Salmonella* colonies per sample were tested using Triple Sugar Iron agar (BD Difco, USA), Lysine Iron agar (BD Difco, USA), Urea agar (BD Difco, USA) and Sulfide Indole Motility (SIM) medium (BD Difco, USA). *Salmonella enterica* subsp. *enterica* serotype Typhimurium ATCC 14028 was used as positive control.

### 2.3 Serotyping

*Salmonella* isolates were serotyped by the French National Reference Laboratory (NRL) for *Salmonella*, hosted by the French Agency for Food, Environment and Occupational Health & Safety (ANSES, Ploufragan Laboratory, France). The White–Kauffmann–Le Minor method, which is based on somatic O antigens and phase 1 and phase 2 flagellar antigens by agglutination tests with antisera, was used (Bio-Rad, Marnes la Coquette, France; Eurobio, Les Ulis, France).

### 2.4 Macrorestriction of chromosomal DNA and genotypic relatedness analysis

Pulsed-field gel electrophoresis (PFGE) was performed by the French NRL for *Salmonella.* The strains were cultured on Plate Count Agar at 37 °C for 24 h. Bacterial DNA was prepared by extracting chromosomal DNA according to the CDC PulseNet standardized procedure for typing *Salmonella* and then digested with the macrorestriction enzyme *Xba*I (Sigma) at 37 °C for 5 h.

Electrophoresis was carried out in a CHEF-DR III system (Bio-Rad), according to the standardized PulseNet protocol (Peters et al., 2003; Ribot et al., 2006). The extracted *Salmonella enterica* subsp*. enterica* serotype Braenderup H9812 was used as a molecular size marker in the PFGE procedure (Hunter et al., 2005). Gels were stained with GelRed as recommended by the supplier (VWR international) and banding patterns were visualized under UV light, using a Quantum CX5 imaging system (Vilber) and Biovision software V 17.06 (Vilber). DNA patterns were analysed with the BioNumerics software (V 7.6.3, Biomerieux) and compared using the algorithms available within the software. A dendrogram was generated using the Dice coefficient and the unweighted pair group method with arithmetic mean (UPGMA), with a 1 % tolerance limit and 1 % optimization. Each PFGE pattern differing by at least one band from a previously recognized type was considered as a new pattern. Each new pattern was given a unique designation, as suggested by Peters et al. (Peters et al., 2003). The diversity of the strains was estimated by calculating the Simpson’s index (ID) (Hunter & Gaston, 1988)with a 95 % confidence interval (Grundmann et al., 2001).

### 2.5 Antimicrobial susceptibility

The antimicrobial resistance of strains was determined using the Kirby-Bauer disk diffusion method, as recommended by the Clinical and Laboratory Standards Institute (CLSI, 2018). *Salmonella enterica* subsp. *enterica* serotype Typhimurium ATCC 14028 was used as positive control.

Briefly, the inoculum was prepared by making a direct broth or saline suspension of isolated colonies selected from an 18- to 24-hour nutrient agar plate, and then the suspension was adjusted to obtain a turbidity equivalent to a standard of 0.5 McFarland. A sterile cotton swab was then dipped in the adjusted solution, turned several times, and then pressed firmly against the inside wall of the tube above the fluid level to remove the excess fluid from the swab. The dried surface of a Mueller Hinton agar plate was then inoculated by streaking the swab over the entire surface of the agar plate. The plate was left stand for 3–5 min to allow for any excess surface moisture to be absorbed before applying the antimicrobial-impregnated disks.

We tested the susceptibility of the isolates to 14 antimicrobials: sulfamethoxazole/trimethoprim (SXT) 25 μg, gentamicin (GEN) 10 μg, ciprofloxacin (CIP) 5 μg, cefotaxime (CTX) 30 μg, tetracycline (TET) 30 μg, streptomycin (S) 10 μg, chloramphenicol (CHN) 30 μg, cefoxitin (CFX) 30 μg, amikacin (AMK) 30 μg, nitrofurantoin (F) 30 μg, azithromycin (AZM) 15 μg, fosfomycin (FOS) 200 μg, ertapenem (ETP) 10 μg, and amoxicillin - clavulanic acid (AMC) 30 μg.

The 14 antimicrobial disks were dispensed onto the surface of the inoculated Mueller Hinton agar plates. Each disk was pressed down to ensure full contact with the agar surface. Plates were finally incubated at 35±2 °C for 24 h. Bacterial resistance to antimicrobials was determined using the clinical breakpoint values from the Clinical and Laboratory Standards Institute (CLSI, 2019).

### 2.6 Detection of the extended-spectrum β-lactamase (ESBL) gene CTX-M

**The β**-lactamase *bla*CTX-M PCR gene **was detected by PCR** on **isolates phenotypically resistant** to cefotaxime. DNA was extracted by the boiling method (Millar et al., 2000). The forward primer used was 5′-ATGTGCAGYACCAGTAARGTKATGGC-3′ and the reverse primer used was 5′-TGGGTRAARTARGTSACCAGAAYCAGCGG-3′ (Hasman et al., 2005). PCR products were visualized in 1 % agarose gel stained with SYBR™ Safe (Invitrogen, Carlsbad, *CA*, USA).

### 2.7 Statistical analysis

All statistical analyses were conducted in R version 4.5.0. The packages used in this study included stats, specifically the functions shapiro.test, chisq.test, and wilcox.test, as well as the car package for leveneTest. The normality of data was assessed using the Shapiro–Wilk test, while the homogeneity of variances was evaluated with Levene’s test. When data were not normally distributed, the Mann–Whitney U test (Wilcoxon rank-sum test) was applied to assess differences between the states and regions considered according to the presence of *Salmonella*. Additionally, Chi squared tests were performed to evaluate associations between the presence of S. enterica in CC and MLNs, as well as across the states and regions considered.

## 3 Results

### 3.1 Occurrence and distribution of Salmonella serotypes in the samples evaluated

Of the 365 pigs tested, 56 (15.3% _CI95%_ [12.2 %-18.4%]) were positive for *S. enterica* in either the cecum (41 CCs out of 365 (11.2% _CI95%_ [8.51-13.95]) or the MLNs (15 MLNs out of 365 (4.1% _CI95%_ [2.40-5.82]), but never in both. *S. enterica* prevalence in the CCs and MLNs was significantly different (Mann Whitney p=0.003). For all samples (CCs and MLNs, n=730), the positivity rate for *S. enterica* was 7.6 % _CI95%_ [6.05-9.29].

Twelve serotypes were identified, the most frequent being the monophasic S. Typhimurium (mST) (28.5% of the isolates), S. Uganda (26.7%) and S. Typhimurium (14.3%) (Table 1). *S*. Uganda and mST were the most prevalent serotypes in the CCs (34.1% and 31.7%, respectively), whereas S. Typhimurium was the most prevalent in MLNs (46.6%).

**Table 1:** Distribution of Salmonella serotypes according to the pig sample type evaluated (cecal contents or mesenteric lymph nodes (MLNs)).

Salmonella serotypes	Total number of isolates [ % out of 56]	Number of isolates in cecal contents [ % out of 41]	Number of isolates in MLNs [ % out of 15]
Monophasic S. Typhimurium	16 [28.5]	13 [31.7]	3 [20.0]
S. Uganda	15 [26.7]	14 [34.1]	1 [6.7]
S. Typhimurium	8 [14.3]	1 [2.4]	7 [46.7]
S. Derby	5 [8.9]	5 [12.2]	-
S. Infantis	4 [7.1]	2 [4.9]	2 [13.3]
S. Meleagridis	2 [3.6]	1 [2.4]	1 [6.7]
S. Amsterdam	1 [1.8]	1 [2.4]	-
S. Falkensee	1 [1.8]	-	1 [6.7]
S. Gaminara	1 [1.8]	1 [2.4]	-
S. Itami	1 [1.8]	1 [2.4]	-
S. Minnesota	1 [1.8]	1 [2.4]	-
S. Muenchen	1[1.8]	1 [2.4]	-
All isolates	56	41	15

The 56 pigs positive for *S. enterica* came from all the states sampled (Table 2). Most positive pigs were from the coastal region, with 62.5 % of positive pigs out of the 24 sampled (Guayas: 71.4 %; Santo Domingo de los Tsachilas: 66.7 %). This percentage was significantly higher than that for pigs coming from the highlands region (12.0 %) (Mann Whitney *p* < 0.001). Among states, the only statistical difference was that between Carchi and Cotopaxi (Mann Whitney *p* < 0.05). *S.* Typhimurium, mST, *S*. Uganda and S. Derby were found in four out of the six states considered in this study (Table 2).

**Table 2:** Geographical distribution of *Salmonella* strains and serotypes.

State	Number of pigs sampled	Number of positive pigs	% of positive pigs per region and state	Serotype	Number of isolates
Highlands region	341	41	12.0		41
Carchi	122	20	16.4	mST	9
				*S*. Uganda	6
				*S*. Typhimurium	3
				*S*. Minnesota	1
				*S*. Derby	1
Pichincha	184	20	10.9	mST	4
				*S*. Uganda	3
				*S*. Typhimurium	3
				*S*. Infantis	3
				*S*. Meleagridis	2
				*S*. Muenchen	1
				*S*. Amsterdam	1
				*S*. Falkensee	1
				*S*. Derby	1
				*S*. Itami	1
Cotopaxi	35	1	1.7	*S*. Typhimurium	1
Coastal region	24	15	62.5		15
Guayas	14	10	71.4	*S*. Uganda	4
				mST	2
				*S*. Derby	2
				*S*. Infantis	1
				*S*. Gaminara	1
Santo Domingo de los Tsachilas	4	4	66.7	mST	2
				*S*. Typhimurium	1
				*S*. Uganda	1
Manabi	6	1	25.0	*S*. Derby	1
TOTAL	365	56	21.9		56

mST: Monophasic S. Typhimurium

### 3.2 Genotypic relatedness

**Fig. 2:**
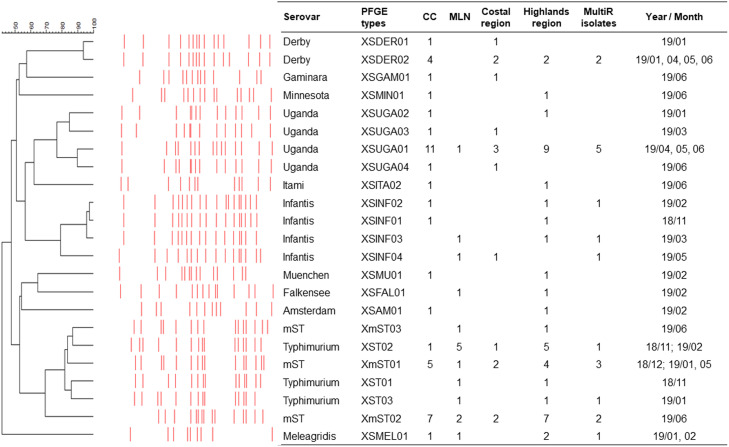
Xbal PFGE profile of*Salmonella* according to sample types (caecum content CC or mesenteric lymph nodes MLN), region (Costal or Highlands). Number of multiresistant isolates and sampling periods (Month/year) are indocated.

The 56 strains were distributed over 22 *Xba*I PFGE genotypes (Figure 2). The overall genetic diversity was high, with a Simpson’s index of 0.910 _CI95%_ [0.87-0.95] but moderate within the three main serotypes: 0.56 _CI95%_ [0.39-0.73] for mST, 0.37 _CI95%_ [0.07-0.67] for *S*. Uganda, and 0.46 _CI95%_ [0.08-0.85] for *S*. Typhimurium.

Five of the genotypes (XSUGA01, XST02, XmST01, XmST02 and XSMEL01) from four different serotypes were found both in the cecum and MLNs, while the others were found only in one type of sample. Five of the genotypes (XSUGA01, XST02, XmST01, XmST02, and XSDER02) were identified in both the coastal and highlands regions (Table 3, supplementary file).

**Table 3:** Geographical distribution of *Salmonella* genotypes.

Serotype	Genotype	Highlands region				Coastal region		
		Carchi	Pichincha	Cotopaxi		Guayas	Santo Domingo de los Tsachilas	Manabi
m*S*T	XmST01	3	1			2		
	XmST02	4	3				2	
	XmST03	1						
*S*. Uganda	XSUGA01	6	3			3		
	XSUGA02	1						
	XSUGA03						1	
	XSUGA04					1		
*S*. Typhimurium	XST01	1						
	XST02	2	2	1			1	
	XST03		1					
*S*. Derby	XSDER01					1		
	XSDER02	1	1			1		1
*S*. Infantis	XSINF01		1					
	XSINF02		1					
	XSINF03		1					
	XSINF04					1		
*S*. Meleagridis	XSMEL01		2					
*S*. Amsterdam	XSAM01		1					
*S*. Falkensee	XSFAL01		1					
*S*. Gaminara	XSGAM01					1		
*S*. Itami	XSITA01		1					
*S*. Minnesota	XSMIN01	1						
*S*. Muenchen	XSMUN01		1					
TOTAL	n = 56	20	20	1		10	4	1

### 3.3 Antimicrobial susceptibility profiles

All strains were susceptible to four out of the 14 antimicrobials tested: amikacin, cefoxitin, ertapenem and ciprofloxacin (Table 4, supplementary file). Eight of the 56 strains were susceptible to all the antimicrobials (4.4 %), whereas the remaining ones (48 out of 56) were resistant to at least one antimicrobial (85.7 %) out of the 14 tested. These 48 strains were distributed over 22 antimicrobial profiles. Of these, 18 strains were resistant to at least three antimicrobials (Table 5) and were therefore considered multiresistant. The two S. Typhimurium strains showed large multiresistance patterns, being resistant to six and eight antimicrobials respectively.

**Table 4:** Percentage of *Salmonella* isolates resistant to each of the 14 antimicrobials tested.

Antimicrobials	Number of resistant isolates [ %]
Tetracycline (TET)	40 [71.4]
Streptomycin (S)	35 [62.5]
Chloramphenicol (CHL)	16 [28.5]
Nitrofurantoin (F)	16 [28.5]
Cefotaxime (CTX)	10 [17.8]
Gentamicin (GEN)	8 [14.2]
Azithromycin (AZM)	6 [10.7]
Sulfamethoxazole/trimethoprim (SXT)	4 [7.1]
Fosfomycin (FOS)	3 [5.3]
Amoxicillin+ clavulanic acid (AMC)	3 [5.3]
Ciprofloxacin (CIP)	0
Cefoxitin (CFX)	0
Amikacin (AMK)	0
Ertapenem (ETP)	0

**Table 5:** Patterns of antimicrobial multiresistance for *Salmonella* isolates (n = 18).

Antimicrobial resistance patterns	Serotype (Number of isolates)
STX, GEN, CTX, TET, S, CHL, F, FOS	*S*. Typhimurium (1*)
GEN, CTX, TET, S, CHL, F	*S*. Typhimurium (1), S. Infantis (1*), S. Uganda (2), S. Derby (1)
STX, CTX, TET, S, CHL, F	*S*. Infantis (2*)
GEN, TET, S, F, AZM	*S*. Uganda (2)
CTX, TET, CHL, AZM	mST (1)
TET, CHL, F, AZM	mST (1)
SXT, TET, S, CHL	*S*. Derby (1)
TET, S, CHL, AZM	mST (1)
TET, S, FOS, AMC	*S*. Uganda (1)
TET, S, F, AMC	mST (1)
TET, S, AMC	mST (1)
TET, S, CHL	*S*. Meleagridis (1)

⁎ ESBL isolate bla-CTM positive. mST: Monophasic *S*. Typhimurium.

A high percentage of strains showed resistance to tetracycline (71.4 %), many to streptomycin (62.5 %) and some to chloramphenicol (28.5 %) (Table 4). Resistance to tetracycline was observed in 93.7 % of the mST strains. Also, all the S. Infantis isolates were resistant to tetracycline and streptomycin.

Resistance to cefotaxime was found in 10 of the isolates (17.4 %) and in the following serotypes: S. Typhimurium (2), S. Infantis (3), S. Uganda (2), S. Derby (1), mST (1) and S. Muenchen (1). The *bla*CTX-M gene was found in four multiresistant strains: three *S*. Infantis and one of S. Typhimurium (Table 5, supplementary file).

## 4 Discussion

The overall prevalence of *S. enterica* found in the present study in pigs slaughtered in Ecuador was 15.3 %. *S. enterica* was found either in the cecum or in the MLNs but, surprisingly, never concomitantly in both types of samples from the same pig. In many studies, concomitant detection of *Salmonella* in both cecum and MLNs has been reported in experimental conditions (M. Cevallos-Almeida et al., 2018) and at slaughterhouse (Magistrali et al., 2008). In our study, the prevalence of *S. enterica* in CCs was significantly higher than that in MLNs. The presence of *Salmonella* in lymph nodes and other tissues represents infection occurring on the farm, rather than recent infection occurring during transport, holding and/or slaughtering (Tay et al., 1989).

*S. enterica* prevalence was also different according to the pig’s origin: 12.0 % in those from the highlands region and 62.5 % in those from the coastal region. This may be due to the climatic conditions specific to each of these regions. The coastal region has tropical and humid climate, with two main seasons, rainy (December to May) and dry (June to November), whereas the highlands region has a more temperate and cooler climate, with a rainy season between October and May. In addition, pigs coming from coastal regions with higher *S. enterica* prevalence have also a longer transport time to reach the slaughterhouse in Quito city. It has been reported that transport duration has a significant impact on the risk of *Salmonella* contamination, due to stress, overcrowding and hygiene conditions (Berends et al., 1997).

*S. enterica* was present in 4.1. % of MLNs (4.1 %). The presence of *Salmonella* in lymphatic tissue represents a risk of contamination because these tissues may be cut during slaughter and processing and can be a potential source of cross-contamination for the facilities (Biasino et al., 2018; Van Damme et al., 2018; Viana et al., 2019). Our results strongly differ from those reported in several studies performed in Brazil, where *Salmonella* prevalence in MLNs reached 35.4 % (Cabral et al., 2017), 43 % (Viana et al., 2019), and 53.48 % (Oliveira et al., 2005). Isolation of *Salmonella* from the ileo-cecal lymph nodes is frequently considered to reflect *Salmonella* prevalence on farms, but short-term exposure in the lairage area can also lead to lymph node positivity (Boughton et al., 2007; Deane et al., 2022; H. S. Hurd et al., 2002). A previous study conducted in the same slaughterhouse indicated that the lairage time ranged from 9 to 12 h (Bucheli & Cevallos-Almeida M., 2022). According to another study, if the lairage time exceeds 12 h, pigs are most likely to be infected with *Salmonella* from the lairage environment compared to pigs held in lairage for approximately one to three hours (Bonardi et al., 2016). Several studies have reported rapid infection with *Salmonella* in pigs at the ceca, feces, and MLN levels two hours after exposure to a contaminated environment (H. Hurd, Gailey et al., 2001; H. Hurd, Mckean et al., 2001; Rostagno et al., 2012).

*Salmonella* is frequently found in the cecum of animals of different species, including swine (M. Cevallos-Almeida et al., 2018; Deane et al., 2022; Gray et al., 1996; Mother et al., 2011). The cecum is the first hospitable environment colonized by *S. enterica*, even before it crosses the colon and invades the lymph nodes (Larsen et al., 2004). In our study, *S. enteria* was isolated from 11.2 % of the cecal contents sampled. This percentage is lower than those found in other Latin American countries such as Argentina and Brazil, where two studies showed higher percentages of 55.9 % and 23.8 %, respectively (Da Silva et al., 2012; Ibar et al., 2009). In European countries such as Ireland and Italy, *Salmonella* was detected in 37.7 % and 34.6 % of pig cecal contents, respectively (Deane et al., 2022; Pesciaroli et al., 2017). Pigs harboring high *Salmonella* loads in the ceca can affect the proportion of carcasses contaminated by *Salmonella* (Pesciaroli et al., 2017), suggesting that highly contaminated pigs play an essential role in carcass contamination. A higher detection rate in the cecal content could be related to the time between *Salmonella* infection and sampling because *Salmonella* can be isolated from the cecum within two hours of exposure to a contaminated environment (Methner et al., 2011).

In the present study, *Salmonella* could not be detected in some of the cecal contents of pigs. Several researchers have observed intermittent shedding for the S. Typhimurium and mST serotypes (Belœil et al., 2003; Boyen et al., 2009; Fernandes et al., 2016; Ivanek et al., 2012). However, this does not explain the absence of *Salmonella* in the ceca of these pigs, as intermittent excretion of *Salmonella* is often linked to continuous carriage, which is expressed punctually by excretion of *Salmonella* in feces. Some authors have indicated that the presence of *Salmonella* in the cecal content favors the presence of *Salmonella* in the distal part of the intestinal tract (colon), thus promoting its shedding (Bernad-Roche et al., 2022; Rostagno et al., 2003; Scherer et al., 2008). In our study, only a few pigs had *S. enterica* in their MLNs. Only two studies in South American countries, one in Brazil and another one in Argentina, have reported *Salmonella* isolation in pig MLNs sampled at slaughterhouses (Possebon et al., 2020; Vico et al., 2020).

In Latin America, the main serotype reported in pork is S. Meleagridis, followed by S. Anatum and *S*. Agona (Ferrari et al., 2019). In Ecuador, the main serotypes reported in pork at retail are mST, S. Infantis and S. Derby (Mejia et al., 2021; Vinueza-Burgos et al., 2023), but there is no information on the serotypes present in pigs at slaughterhouse. In the present work, we identified a wide variety of serotypes, with a greater diversity in the pigs from the highlands region, probably due to a higher number of positive pigs from this region. The most frequently isolated common serotypes in the present investigation were mST, *S*. Typhimurium and *S*. Derby, with percentages of 28.6 %, 14.3 % and 8.9 %, respectively. These serotypes are the most frequently reported in the European Union and the United States (EFSA-ECDC, 2023; Soliani et al., 2023). In particular, mST is a serotype that has emerged in humans worldwide (De la Torre et al., 2003; Napoleoni et al., 2023; Soyer et al., 2009; Switt et al., 2009), and pigs are considered major carriers (Hauser et al., 2010; Ruggeri et al., 2015). In the European Union, the mST sequence type ST34 has become a global pandemic clone in humans (Petrovska et al., 2016). In Ecuador, mST has been recently isolated from pork sold in public markets (53.3 % of isolated strains), confirming that this serotype is circulating in the pig sector in Ecuador (Vinueza-Burgos et al., 2023).

Previous research has indicated that mST strains show considerable subtype diversity, even among samples from a single country. This heterogeneity implies greater adaptability, which may contribute to the success of this serotype (Shi et al., 2025; Zamperini et al., 2007). Also, the spread of mST strains in swine populations around the world is probably linked to the selective advantage offered by multi-drug-resistant strains associated with stable genetic elements, also carrying virulence determinants within bacterial lineages well adapted to the porcine host and widespread in human infections resulting from contaminated pork meat (EFSA-ECDC, 2017; Marin et al., 2020). However, the involvement of this serotype in human salmonellosis in Ecuador is unknown, as official epidemiological reports only describe *Salmonella* spp. (Ministerio de Salud Pública del Ecuador, 2021; Vinueza-Burgos et al., 2023). The presence of this emerging serotype in pigs probably poses a risk to humans in Ecuador, especially because it is generally associated with resistance to antibiotics. The other serotypes isolated in this study were *S*. Typhimurium and *S*. Infantis. *S*. Typhimurium has been frequently reported in many South American countries as the main serotype found in swine (Bersot et al., 2019; Ferrari et al., 2019; Possebon et al., 2020; Vico et al., 2020). In Ecuador, *S*. Infantis has been extensively studied and has been identified in various samples of animal origin such as chicken carcasses, chicken cecum content, layer hen farms and pork sold in public markets (Mejía et al., 2020; Mejia et al., 2021; Salazar et al., 2019).

Various uncommon serotypes, such as *S*. Uganda, were also found in the present study. This serotype was found in 26.7 % of the isolates, making it the second most frequent serotype found in this study. *S*. Uganda has rarely been described in pigs. In previous studies, this serotype was found in 3.9 % of the isolates from pigs slaughtered in Canada (Sanchez-Maldonado et al., 2017) and in 4.3 % of the isolates from pork samples at retail in China(Miao et al., 2022). It has been more widely reported in poultry, turkeys, and cattle in the United States and Canada (Murray et al., 2023; Sodagari et al., 2023; S. Zhao et al., 2007). In France, data collected by the ANSES *Salmonella* network from 2001 to 2024 showed that a majority of S. Uganda strains were also isolated from poultry (151 out of 230; 65.5 %), mainly from French overseas department and region (94.7 % from Reunion Island and French Guiana) (ANSES *Salmonella* network; personal communication).

In Ecuador, 95 % of the pig production is family-type (ASPE-ECUADOR, 2017). Although farms with several animal productions have not been reported in this country, family farms generally have chickens, pigs, ducks and some cattle (Bastidas-Caldes et al., 2023). In addition, many smallholdings do not have enclosures for their animals, so chicken, pigs and other animals can be exposed to bacteria present in the farm environment, on surrounding farms and from nearby commercial operations (Amato et al., 2023; Chavez-Lindell et al., 2022). The use of manure and slurry to fertilize the soil and the movement of animals reared outdoors are also sources of the spread of zoonotic enteric pathogens in the environment (Amato et al., 2023; Lowenstein et al., 2020). This situation may explain the variety of rare serotypes found in this study and the description of similar serotypes in different animal productions.

In the present study, we found 22 PFGE profiles, 18 for the 41 strains isolated from pigs reared in the states of the highland’s region and 11 for the 15 strains isolated from pigs reared in the states of the coastal region. Pigs coming from the coastal region are mainly from family farms carried by merchants called “*introductores*” (Spanish term meaning “introducers”), who prefer to buy pigs in the surrounding cities and states. These merchants also select and transport pigs from an important market in the coastal state of Santo Domingo de los Tsachilas. The diversity in the origin of pigs in the coastal region could explain their greater genotypic diversity.

Different PFGE profiles were observed within the same serotype, particularly for S. Infantis and S. Uganda, followed by *S*. Typhimurium and mST. As already described (Siddi et al., 2021), PFGE profiles of a same serotype cluster together in the general dendrogram, with the exception of *S.* Typhimurium and mST PFGE profiles, which are grouped within the same cluster.

In our study, S. Derby isolates showed poor PFGE diversity, although they were from pigs raised in six different states of two different regions and were isolated on four different dates. The limited diversity of S. Derby isolates has already been described (Kerouanton et al., 2013). This serotype is considered polyphyletic (Sévellec et al., 2018). When comparing the PFGE profiles here obtained with PFGE profiles isolated from pigs in France, we observed that the two PFGE profiles found for S. Derby in the present study were similar (with >96 % and 86 % of genetic similarity, respectively) to a profile widely observed in the French pig production sector as well as in French pork and, overall, in humans (Kerouanton et al., 2013). Some French isolates with this genotype were sequenced and showed to belong to the sequence type ST40 (data not shown).

It is also important to mention that the XmST02 profile was found on the same day of slaughter both in the CCs and MLNs from two different pigs. Contamination between these pigs may have occurred during the lairage period. This XmST02 profile was similar to the PFGE profile already observed in France in 2007 from conventional pork with >95 % of genetic similarity (Denis et al., 2009) and in 2012 from organic pork (Denis et al., 2013). These French XmST02 isolates belonged to the sequence type ST34. This sequence type is very common in mST isolates (Teunis et al., 2025) and a recent source attribution study performed in Europe confirmed infection of humans with pig isolates of this sequence type (data not shown). We also observed a similar PFGE profile XST01 (corresponding to sequence type ST19) between Ecuadorian isolates and French isolates detected in a French pig slaughterhouse in 2007 (Denis et al., 2009). PFGE remains a practical tool for tracing *Salmonella* in a slaughterhouse but has limitations for comparing PFGE profiles obtained from other studies and countries. In this study, the comparison with French strains could be made because strains in this study were typed in the same laboratory. It would be interesting to complete this study by sequencing the Ecuadorian strains to compare their sequence types with others from various countries.

The antimicrobial resistance tests carried out on *S. enterica* isolates in the present study indicated that 85.7 % of them were resistant to at least one of the 14 antibiotics tested. Our results are consistent with those found for *Salmonella* isolated from pork shipped to retail and public markets in the city of Quito (Vinueza-Burgos et al., 2023). About 71.4 % of the strains were found to be resistant to tetracycline and 23.3 % to chloramphenicol. Similar percentages were also observed for *Salmonella* isolated from chicken carcasses in Ecuadorian slaughterhouses (Amancha et al., 2023). The high percentage of resistance to tetracycline can be explained by the fact that tetracycline is the second most used antibiotic in Ecuador (Aguilar et al., 2018) and was the most imported antibiotic by Ecuador in 2019 for veterinary medical use. In addition, 62.5 % of our strains were resistant to streptomycin. This percentage is lower than that observed for S. Infantis (90.4 %) and higher than that observed for S. Enteritidis (22.2 %) isolated from broilers at slaughter in Ecuador (Vinueza-Burgos et al., 2016). Resistance to cefotaxime was low (17.9 %). This value is lower than cefotaxime resistance values previously reported in humans (33.3 % of the strains), chicken carcasses (82.9 %) and poultry houses (94.4 %) in Ecuador (Mejía et al., 2020).

The *bla*CTX-M gene was detected by PCR in eight of the strains. ESBL genes in *Salmonella* isolated from pigs have been recently reported in Belgium, China and France (de Jong et al., 2014; Sévellec et al., 2018; X. Zhao et al., 2017). These genes confer resistance to third-generation cephalosporins, which are antibiotics used in human medicine (Bush & Bradford, 2020; World Health Organization (WHO), 2024). Moreover, the localization of these genes in plasmids facilitates their horizontal transfer between bacteria, including those that colonize humans, which constitutes a public health risk (Carattoli, 2013).

Eighteen out of the 56 strains (32.1 %) had multiresistance antibiotic patterns, particularly *S*. Typhimurium (found to be resistant to up to eight antibiotics), followed by *S*. Infantis. In swine, multi-drug resistant strains are common, particularly S. Typhimurium and mST, which represent the two main serotypes associated with swine production in several countries (EFSA-ECDC, 2021; Galán-Relaño et al., 2022).

In Ecuador, despite the ban of certain antibiotics such as chloramphenicol and colistin in animal production (AGROCALIDAD, 2016, 2020; Bastidas-Caldes et al., 2023), farmers generally purchase and administer antibiotics themselves to treat their animals for economic reasons (Martínez et al., 2023), but often stop treatment too early after clinical signs disappear, a fact that forces them to restart treatment if symptoms reappear (Martínez et al., 2023). Unfortunately, these practices promote antibiotic resistance in pathogens, including *Salmonella*.

This study has some limitations that should be considered when interpreting the results. First, the data were collected from a single slaughterhouse, although the largest in the region. This choice provides a representative picture of the sector's core activity but may not fully reflect the diversity of practices or conditions that could be observed in other smaller establishments or those with different characteristics.

Finally, the use of complementary tools, such as cross-referencing data with meteorological information, could offer new insights and more detailed interpretations. For example, weather conditions can influence certain aspects of the process studied, and their integration into future analysis could significantly enrich the results obtained.

## 5 Conclusions

This study established for the first time the prevalence of *Salmonella enterica* in pigs in a slaughterhouse in Ecuador. The high rate of contaminated ceca and the wide variety of serotypes and genotypes observed suggest that contamination occurs mainly at the pre-slaughter stage, where cross-contamination between pigs at farms, during transport and lairage can occur. The main serotype found, monophasic *S*. Typhimurium, is common in the swine industry, whereas the second serotype, *S*. Uganda, is less common and specific for this study. The multidrug resistance profiles observed among the isolates highlight the critical necessity to monitor the spread of antibiotic resistance in pig farms in Ecuador. This large-scale study in one slaughterhouse provides a solid basis for extending this investigation to a larger number of slaughterhouses in Ecuador, including small-scale slaughterhouses, also taking into account the geographical and climatic factors specific to this country. This will provide additional data on the serotypes, genotypes and antibiotic resistance profiles of *Salmonella enterica* circulating in Ecuador.

## Funding

This research was funded by the Research Directorate of the Central University of Ecuador (Project No 2–2016). The funders had no role in the study design, data collection and analysis, decision to publish, or preparation of the manuscript.

For the purpose of Open Access, the authors have applied a CC BY public copyright licence to any Author Accepted Manuscript (AAM) version arising from this submission*.*

## Data availability

The datasets used and/or analyzed during the current study are available from the corresponding author on reasonable request.

Ethics in Publishing Statement

This research presents an accurate account of the work performed, all data presented are accurate and methodologies detailed enough to permit others to replicate the work.

This manuscript represents entirely original works and or if work and/or words of others have been used, that this has been appropriately cited or quoted and permission has been obtained where necessary.

This material has not been published in whole or in part elsewhere.

The manuscript is not currently being considered for publication in another journal.

That generative AI and AI-assisted technologies have not been utilized in the writing process or if used, disclosed in the manuscript the use of AI and AI-assisted technologies and a statement will appear in the published work.

That generative AI and AI-assisted technologies have not been used to create or alter images unless specifically used as part of the research design where such use must be described in a reproducible manner in the methods section.

All authors have been personally and actively involved in substantive work leading to the manuscript and will hold themselves jointly and individually responsible for its content.

## CRediT authorship contribution statement

**M. Cevallos-Almeida:** Writing – review & editing, Writing – original draft, Validation, Resources, Project administration, Investigation, Funding acquisition, Formal analysis, Data curation, Conceptualization. **C. Gómez:** Methodology, Investigation. **B. Cajas:** Investigation. **A. Almachi:** Investigation. **V. Rose:** Methodology, Investigation. **M. Denis:** Writing – review & editing, Writing – original draft, Validation, Supervision, Formal analysis, Data curation. **A. Kerouanton:** Writing – review & editing, Writing – original draft, Validation, Supervision, Project administration, Investigation, Formal analysis, Data curation, Conceptualization.

## Declaration of competing interest

The authors declare that they have no known competing financial interests or personal relationships that could have appeared to influence the work reported in this paper.

## Appendix Supplementary materials

Image, application 1
