# Supplementary material for: Salmonella in pigs slaughtered in Ecuador: prevalence, serotypes, genotypes and antibiotic resistance
Source: Vet Anim Sci. 2025 Nov 22;30:100548. doi: 10.1016/j.vas.2025.100548 (PMC12702343; doi:10.1016/j.vas.2025.100548)
Supplement: Supplementary file 1 [file mmc1.docx]

| **Isolate_No** | **Region** | **State** | **Sample** | **Serotype** | **Genotype** | **SXT** | **GEN** | **CIP** | **CTX** | | **TET** | **S** | **CHL** | **CFX** | **AMK** | **F** | | **AZM** | **FOS** | **ETP** | | **AMC** | **Antibioresistance_profile** | | | | | | **CTX-M gene** |  |  |  |  |  |  |  |  |  |  |  |  |  |  |  |  |  |  |  |  |  |
| --- | --- | --- | --- | --- | --- | --- | --- | --- | --- | --- | --- | --- | --- | --- | --- | --- | --- | --- | --- | --- | --- | --- | --- | --- | --- | --- | --- | --- | --- | --- | --- | --- | --- | --- | --- | --- | --- | --- | --- | --- | --- | --- | --- | --- | --- | --- | --- | --- | --- | --- |
| SLB18BC95 | Coastal | Manabí | Caecum | Derby | XSDER02 | R | S | S | S | | R | R | R | S | S | S | | S | S | S | | S | SXT, TET, S, CHL | | | | | | nd |  |  |  |  |  |  |  |  |  |  |  |  |  |  |  |  |  |  |  |  |  |
| SLB19BC62 | Coastal | Guayas | Caecum | Derby | XSDER01 | S | S | S | S | | R | S | S | S | S | S | | S | S | S | | S | TET | | | | | | nd |  |  |  |  |  |  |  |  |  |  |  |  |  |  |  |  |  |  |  |  |  |
| SLB19BC273 | Coastal | Guayas | Caecum | Derby | XSDER02 | S | S | S | S | | S | R | S | S | S | S | | S | S | S | | S | S | | | | | | nd |  |  |  |  |  |  |  |  |  |  |  |  |  |  |  |  |  |  |  |  |  |
| SLB19BC565 | Coastal | Guayas | Caecum | Gaminara | XSGAM01 | S | S | S | S | | R | R | S | S | S | S | | S | S | S | | S | TET, S | | | | | | nd |  |  |  |  |  |  |  |  |  |  |  |  |  |  |  |  |  |  |  |  |  |
| SLB19BC600 | Coastal | Guayas | Caecum | mST | XmST01 | S | S | S | S | | R | R | R | S | S | S | | R | S | S | | S | TET, S, CHL, AZM | | | | | | nd |  |  |  |  |  |  |  |  |  |  |  |  |  |  |  |  |  |  |  |  |  |
| SLB19BC524 | Coastal | STD | Caecum | mST | XmST02 | S | S | S | S | | R | R | S | S | S | S | | S | S | S | | S | TET, S | | | | | | nd |  |  |  |  |  |  |  |  |  |  |  |  |  |  |  |  |  |  |  |  |  |
| SLB19BC527 | Coastal | STD | Caecum | mST | XmST02 | S | S | S | S | | R | R | S | S | S | S | | S | S | S | | S | TET, S | | | | | | nd |  |  |  |  |  |  |  |  |  |  |  |  |  |  |  |  |  |  |  |  |  |
| SLB19BC127 | Coastal | STD | Caecum | Uganda | XSUGA03 | S | S | S | S | | R | S | S | S | S | S | | S | S | S | | S | TET | | | | | | nd |  |  |  |  |  |  |  |  |  |  |  |  |  |  |  |  |  |  |  |  |  |
| SLB19BC370 | Coastal | Guayas | Caecum | Uganda | XSUGA01 | S | R | S | S | | R | R | S | S | S | R | | R | S | S | | S | GEN, TET, S, F, AZM | | | | | | nd |  |  |  |  |  |  |  |  |  |  |  |  |  |  |  |  |  |  |  |  |  |
| SLB19BC604 | Coastal | Guayas | Caecum | Uganda | XSUGA01 | S | S | S | S | | R | R | S | S | S | S | | S | R | S | | R | TET, S, FOS, AMC | | | | | | nd |  |  |  |  |  |  |  |  |  |  |  |  |  |  |  |  |  |  |  |  |  |
| SLB19BC557 | Coastal | Guayas | Caecum | Uganda | XSUGA01 | S | S | S | S | | R | R | S | S | S | S | | S | S | S | | S | TET, S | | | | | | nd |  |  |  |  |  |  |  |  |  |  |  |  |  |  |  |  |  |  |  |  |  |
| SLB19BC570 | Coastal | Guayas | Caecum | Uganda | XSUGA04 | S | S | S | S | | R | R | S | S | S | S | | S | S | S | | S | TET, S | | | | | | nd |  |  |  |  |  |  |  |  |  |  |  |  |  |  |  |  |  |  |  |  |  |
| SLB19BC606 | Coastal | Guayas | MLN | Infantis | XSINF04 | R | S | S | R | | R | R | R | S | S | R | | S | S | S | | S | STX, CTX, TET, S, CHL, F | | | | | | Present |  |  |  |  |  |  |  |  |  |  |  |  |  |  |  |  |  |  |  |  |  |
| SLB19BC522 | Coastal | Guayas | MLN | mST | XmST01 | S | S | S | S | | R | S | R | S | S | S | | S | S | S | | S | TET, CHL | | | | | | nd |  |  |  |  |  |  |  |  |  |  |  |  |  |  |  |  |  |  |  |  |  |
| SLB19BC104 | Coastal | STD | MLN | Typhimurium | XST02 | S | S | S | S | | S | S | S | S | S | S | | S | S | S | | S | Susceptible | | | | | | nd |  |  |  |  |  |  |  |  |  |  |  |  |  |  |  |  |  |  |  |  |  |
| SLB19BC86 | Highlands | Pichincha | Caecum | Amsterdam | XSAMS01 | S | S | S | S | | S | S | S | S | S | S | | S | S | S | | S | Susceptible | | | | | | nd |  |  |  |  |  |  |  |  |  |  |  |  |  |  |  |  |  |  |  |  |  |
| SLB19BC597 | Highlands | Carchi | Caecum | Derby | XSDER02 | S | S | S | S | | S | S | S | S | S | S | | S | S | S | | S | Susceptible | | | | | | nd |  |  |  |  |  |  |  |  |  |  |  |  |  |  |  |  |  |  |  |  |  |
| SLB19BC572 | Highlands | Pichincha | Caecum | Derby | XSDER02 | S | R | S | R | | R | R | R | S | S | R | | S | S | S | | S | GEN, CTX, TET, S, CHL, F | | | | | | Absent |  |  |  |  |  |  |  |  |  |  |  |  |  |  |  |  |  |  |  |  |  |
| SLB18BC60 | Highlands | Pichincha | Caecum | Infantis | XSINF01 | S | S | S | S | | R | R | S | S | S | S | | S | S | S | | S | TET, S | | | | | | nd |  |  |  |  |  |  |  |  |  |  |  |  |  |  |  |  |  |  |  |  |  |
| SLB19BC72 | Highlands | Pichincha | Caecum | Infantis | XSINF02 | S | R | S | R | | R | R | R | S | S | R | | S | S | S | | S | GEN, CTX, TET, S, CHL, F | | | | | | Present |  |  |  |  |  |  |  |  |  |  |  |  |  |  |  |  |  |  |  |  |  |
| SLB19BC582 | Highlands | Pichincha | Caecum | Itami | XSITA02 | S | S | S | S | | S | R | S | S | S | S | | S | S | S | | S | S | | | | | | nd |  |  |  |  |  |  |  |  |  |  |  |  |  |  |  |  |  |  |  |  |  |
| SLB19BC58 | Highlands | Pichincha | Caecum | Meleagridis | XSMEL01 | S | S | S | S | | R | S | S | S | S | S | | S | S | S | | S | TET | | | | | | nd |  |  |  |  |  |  |  |  |  |  |  |  |  |  |  |  |  |  |  |  |  |
| SLB19BC397 | Highlands | Carchi | Caecum | Minnesota | XSMIN01 | S | S | S | S | | S | R | S | S | S | S | | S | S | S | | S | S | | | | | | nd |  |  |  |  |  |  |  |  |  |  |  |  |  |  |  |  |  |  |  |  |  |
| SLB18BC79 | Highlands | Pichincha | Caecum | mST | XmST01 | S | S | S | S | | R | S | R | S | S | S | | S | S | S | | S | TET, CHL | | | | | | nd |  |  |  |  |  |  |  |  |  |  |  |  |  |  |  |  |  |  |  |  |  |
| SLB18BC88 | Highlands | Carchi | Caecum | mST | XmST01 | S | S | S | S | | R | S | R | S | S | R | | R | S | S | | S | TET, CHL, F, AZM | | | | | | nd |  |  |  |  |  |  |  |  |  |  |  |  |  |  |  |  |  |  |  |  |  |
| SLB19BC01 | Highlands | Carchi | Caecum | mST | XmST01 | S | S | S | S | | R | S | R | S | S | S | | S | S | S | | S | CHL | | | | | | nd |  |  |  |  |  |  |  |  |  |  |  |  |  |  |  |  |  |  |  |  |  |
| SLB19BC602 | Highlands | Carchi | Caecum | mST | XmST01 | S | S | S | R | | R | S | R | S | S | S | | R | S | S | | S | CTX, TET,CHL, AZM | | | | | | Absent |  |  |  |  |  |  |  |  |  |  |  |  |  |  |  |  |  |  |  |  |  |
| SLB18BC96 | Highlands | Carchi | Caecum | mST | XmST02 | S | S | S | S | | R | R | S | S | S | R | | S | S | S | | R | TET, S, F, AMC | | | | | | nd |  |  |  |  |  |  |  |  |  |  |  |  |  |  |  |  |  |  |  |  |  |
| SLB19BC515 | Highlands | Carchi | Caecum | mST | XmST02 | S | S | S | S | | R | R | S | S | S | S | | S | S | S | | S | TET, S | | | | | | nd |  |  |  |  |  |  |  |  |  |  |  |  |  |  |  |  |  |  |  |  |  |
| STD = Santo Domingo de Los Tsáchilas; R: Resistant; S : Sensitive; nd : not done | | | | | | | | | | | | | | | | | | | | | | | | | | | | | |  |  |  |  |  |  |  |  |  |  |  |  |  |  |  |  |  |  |  |  |  |
| **Isolate_No** | **Region** | **State** | **Sample** | **Serotype** | **Genotype** | **SXT** | **GEN** | **CIP** | **CTX** | | **TET** | **S** | **CHL** | **CFX** | **AMK** | **F** | | **AZM** | **FOS** | **ETP** | | **AMC** | **Antibioresistance_profile** | | | | | | **CTX-M gene** |  |  |  |  |  |  |  |  |  |  |  |  |  |  |  |  |  |  |  |  |  |
| SLB19BC516 | Highlands | Carchi | Caecum | mST | XmST02 | S | S | S | S | | R | R | S | S | S | S | | S | S | S | | S | TET, S | | | | | | nd |  |  |  |  |  |  |  |  |  |  |  |  |  |  |  |  |  |  |  |  |  |
| SLB19BC519 | Highlands | Carchi | Caecum | mST | XmST02 | S | S | S | S | | R | R | S | S | S | S | | S | S | S | | R | TET, S, AMC | | | | | | nd |  |  |  |  |  |  |  |  |  |  |  |  |  |  |  |  |  |  |  |  |  |
| SLB19BC529 | Highlands | Pichincha | Caecum | mST | XmST02 | S | S | S | S | | R | R | S | S | S | S | | S | S | S | | S | TET, S | | | | | | nd |  |  |  |  |  |  |  |  |  |  |  |  |  |  |  |  |  |  |  |  |  |
| SLB19BC531 | Highlands | Pichincha | Caecum | mST | XmST02 | S | S | S | S | | R | R | S | S | S | S | | S | S | S | | S | TET, S | | | | | | nd |  |  |  |  |  |  |  |  |  |  |  |  |  |  |  |  |  |  |  |  |  |
| SLB19BC532 | Highlands | Pichincha | Caecum | mST | XmST02 | S | S | S | S | | R | R | S | S | S | S | | S | S | S | | S | TET, S | | | | | | nd |  |  |  |  |  |  |  |  |  |  |  |  |  |  |  |  |  |  |  |  |  |
| SLB19BC09 | Highlands | Carchi | Caecum | mST | XmST03 | S | S | S | S | | S | S | S | S | S | R | | R | S | S | | S | F, AZM | | | | | | nd |  |  |  |  |  |  |  |  |  |  |  |  |  |  |  |  |  |  |  |  |  |
| SLB19BC68 | Highlands | Pichincha | Caecum | Muenchen | XSMUN01 | S | S | S | R | | S | S | S | S | S | S | | S | S | S | | S | CTX | | | | | | Absent |  |  |  |  |  |  |  |  |  |  |  |  |  |  |  |  |  |  |  |  |  |
| SLB19BC106 | Highlands | Cotopaxi | Caecum | Typhimurium | XST02 | R | R | S | R | | R | R | R | S | S | R | | S | R | S | | S | SXT, GEN, CTX, TET, S, CHL, F, FOS | | | | | | Present |  |  |  |  |  |  |  |  |  |  |  |  |  |  |  |  |  |  |  |  |  |
| SLB19BC38 | Highlands | Carchi | Caecum | Uganda | XSUGA02 | S | S | S | S | | R | S | S | S | S | R | | S | S | S | | S | TET, F | | | | | | nd |  |  |  |  |  |  |  |  |  |  |  |  |  |  |  |  |  |  |  |  |  |
| SLB19BC552 | Highlands | Carchi | Caecum | Uganda | XSUGA01 | S | S | S | S | | R | R | S | S | S | S | | S | S | S | | S | TET, S | | | | | | nd |  |  |  |  |  |  |  |  |  |  |  |  |  |  |  |  |  |  |  |  |  |
| SLB19BC605 | Highlands | Carchi | Caecum | Uganda | XSUGA01 | S | S | S | S | | R | R | S | S | S | S | | S | S | S | | S | TET, S | | | | | | nd |  |  |  |  |  |  |  |  |  |  |  |  |  |  |  |  |  |  |  |  |  |
| SLB19BC371 | Highlands | Carchi | Caecum | Uganda | XSUGA01 | S | S | S | S | | S | S | S | S | S | R | | S | S | S | | S | F | | | | | | nd |  |  |  |  |  |  |  |  |  |  |  |  |  |  |  |  |  |  |  |  |  |
| SLB19BC373 | Highlands | Pichincha | Caecum | Uganda | XSUGA01 | S | S | S | S | | S | S | S | S | S | S | | S | S | S | | S | Susceptible | | | | | | nd |  |  |  |  |  |  |  |  |  |  |  |  |  |  |  |  |  |  |  |  |  |
| SLB19BC375 | Highlands | Pichincha | Caecum | Uganda | XSUGA01 | S | S | S | S | | R | R | S | S | S | S | | S | S | S | | S | TET, S | | | | | | nd |  |  |  |  |  |  |  |  |  |  |  |  |  |  |  |  |  |  |  |  |  |
| SLB19BC378 | Highlands | Carchi | Caecum | Uganda | XSUGA01 | S | R | S | S | | R | R | S | S | S | R | | R | S | S | | S | GEN, TET, S, F, AZM | | | | | | nd |  |  |  |  |  |  |  |  |  |  |  |  |  |  |  |  |  |  |  |  |  |
| SLB19BC510 | Highlands | Pichincha | Caecum | Uganda | XSUGA01 | S | S | S | S | | R | R | S | S | S | S | | S | S | S | | S | TET, S | | | | | | nd |  |  |  |  |  |  |  |  |  |  |  |  |  |  |  |  |  |  |  |  |  |
| SLB19BC555 | Highlands | Carchi | Caecum | Uganda | XSUGA01 | S | R | S | R | | R | R | R | S | S | R | | S | S | S | | S | GEN, CTX, TET, S, CHL, F | | | | | | Absent |  |  |  |  |  |  |  |  |  |  |  |  |  |  |  |  |  |  |  |  |  |
| SLB19BC103 | Highlands | Pichincha | MLN | Falkensee | XSFAL01 | S | S | S | S | | S | S | S | S | S | S | | S | S | S | | S | Susceptible | | | | | | nd |  |  |  |  |  |  |  |  |  |  |  |  |  |  |  |  |  |  |  |  |  |
| SLB19BC137 | Highlands | Pichincha | MLN | Infantis | XSINF03 | R | S | S | R | | R | R | R | S | S | R | | S | S | S | | S | SXT, CTX, TET, S, CHL, F | | | | | | Present |  |  |  |  |  |  |  |  |  |  |  |  |  |  |  |  |  |  |  |  |  |
| SLB19BC73 | Highlands | Pichincha | MLN | Meleagridis | XSMEL01 | S | S | S | S | | R | R | R | S | S | S | | S | S | S | | S | CTX, TET, S | | | | | | nd |  |  |  |  |  |  |  |  |  |  |  |  |  |  |  |  |  |  |  |  |  |
| SLB18BC50 | Highlands | Carchi | MLN | Typhimurium | XST01 | S | S | S | S | | S | S | S | S | S | S | | S | S | S | | S | Susceptible | | | | | | nd |  |  |  |  |  |  |  |  |  |  |  |  |  |  |  |  |  |  |  |  |  |
| SLB18BC71 | Highlands | Pichincha | MLN | Typhimurium | XST02 | S | S | S | S | | S | S | S | S | S | R | | S | S | S | | S | FOS | | | | | | nd |  |  |  |  |  |  |  |  |  |  |  |  |  |  |  |  |  |  |  |  |  |
| SLB18BC73 | Highlands | Pichincha | MLN | Typhimurium | XST02 | S | S | S | S | | S | S | S | S | S | S | | S | S | S | | S | Susceptible | | | | | | nd |  |  |  |  |  |  |  |  |  |  |  |  |  |  |  |  |  |  |  |  |  |
| SLB19BC64 | Highlands | Pichincha | MLN | Typhimurium | XST03 | S | R | S | R | | R | R | R | S | S | R | | S | S | S | | S | GEN, CTX, TET, S, CHL, F | | | | | | Absent |  |  |  |  |  |  |  |  |  |  |  |  |  |  |  |  |  |  |  |  |  |
| SLB19BC99 | Highlands | Carchi | MLN | Typhimurium | XST02 | S | S | S | S | | S | S | S | S | S | S | | S | S | S | | S | Susceptible | | | | | | nd |  |  |  |  |  |  |  |  |  |  |  |  |  |  |  |  |  |  |  |  |  |
| SLB19BC102 | Highlands | Carchi | MLN | Typhimurium | XST02 | S | S | S | S | | S | R | S | S | S | S | | S | R | S | | S | S, FOS | | | | | | nd |  |  |  |  |  |  |  |  |  |  |  |  |  |  |  |  |  |  |  |  |  |
| SLB19BC372 | Highlands | Carchi | MLN | Uganda | XSUGA01 | S | R | S | R | | R | R | R | S | S | R | | S | S | S | | S | GEN, CTX, TET, S, CHL, F | | | | | | Absent |  |  |  |  |  |  |  |  |  |  |  |  |  |  |  |  |  |  |  |  |  |
| STD = Santo Domingo de Los Tsáchilas; R: Resistant; S : Sensitive; nd : not done | | | | | | | | | | | | | | | | | | | | | | | | | | | | | | |  |  |  | |  |  |  |  | |  | | | | |  |  |  |  |  |  |
|  | | | | | | | | | |  | | | | | | |  | | | |  | | |  |  |  |  |  | | | | | |  | | | | |  | |  |  |  |  | |  |  |  |  |  |
